# PD42 - Is rituximab a trigger for persistent hypogammaglobulinemia in idiopathic nephrotic syndrome?

**DOI:** 10.1186/2045-7022-4-S1-P42

**Published:** 2014-02-28

**Authors:** Juan Enrique Trujillo, Montserrat Bosque, Òscar Asensio, Adrián Ranera, Juan Cristobal Rojo, Mireia Vilella, Elisabet Guijarro, Xavier Domingo, Laura Valdesoiro, Helena Larramona

**Affiliations:** 1 Corporació Sanitària Parc Taulí, Sabadell, Barcelona, Spain

## 

Rituximab (RTX) is a monoclonal antibody; recently it has been use as a new treatment strategy in patients with high-degree steroid-dependent nephrotic syndrome. It provokes a lymphocyte B depletion in some cases that interacts with Immunoglobulins creating decreased plasma IgG levels and hypogammaglobulinemia.

We report the case of a 2.5 year old male diagnosed of idiopathic nephrotic syndrome. He presented resistance to systemic steroids as well as second line treatments (cyclophosphamide and mycophenolate). Patient started with intravenous infussion of rituximab at 375mg/m^2^. Baseline analysis before RTX treatment evidenced low IgG levels, although these values are not conclusive because they were taken during nephrotic state. Twenty days later, the patient presented severe hypogammaglobulinemia with IgG levels of 149 mg/dL, without nephrotic proteinuria. We decided to start intravenous gammaglobulin (IVIG) every 4-6 weeks. Analysis showed low levels of lymphocytes (predominantly B type) four days after initiated the treatment. Follow up demonstrated complete recovery of lymphocyte count two years later. After 3 years of RTX administration the patient continues to have hypogammaglobulimenia, requiring IVIG until now.

## Comments

The cause of immunodeficiency in this patient has not yet been established. Is it because of the nephrotic syndrome itself? An adverse effect of the drug? Maybe a pre-existing primary immunodeficiency?

Use of RTX has reported cases of hypogammaglobulinemia values usually normalizing after 11 months; persistent hypogammaglobulinemia is not a common side effect. We only found one series describing the association of persistent hypogammaglobulinemia in nephrotic patients treated with RTX, suggesting a certain predisposition in patients with low baseline IgG levels previous to the treatment with this monoclonal antibody.

Starting a full immune panel previous to treatment with RTX could be a good way to predict possible persistent hypogammaglobulinemia and to rule out immunologic pathology before making any therapeutic decision.

